# Elucidation of Antimicrobial Silver Sulfadiazine (SSD) Blend/Poly(3-Hydroxybutyrate-*co*-4-Hydroxybutyrate) Immobilised with Collagen Peptide as Potential Biomaterial

**DOI:** 10.3390/polym12122979

**Published:** 2020-12-14

**Authors:** Sevakumaran Vigneswari, Tana Poorani Gurusamy, H. P. S. Abdul Khalil, Seeram Ramakrishna, Al-Ashraf Abdullah Amirul

**Affiliations:** 1 Faculty of Science and Marine Environment, Universiti Malaysia Terengganu, Kuala Nerus 21030, Terengganu, Malaysia; vicky@umt.edu.my; 2 School of Biological Sciences, Universiti Sains Malaysia, Penang 11800, Malaysia; purani_guru@yahoo.com; 3 School of Industrial Technology, Universiti Sains Malaysia, Penang 11800, Malaysia; akhalilhps@gmail.com; 4 Center for Nanofibers and Nanotechnology, Department of Mechanical Engineering, National University of Singapore, Singapore 117581, Singapore; seeram@nus.edu.sg; 5 Centre for Chemical Biology, Universiti Sains Malaysia, Bayan Lepas, Penang 11900, Malaysia; 6 Malaysian Institute of Pharmaceuticals and Nutraceuticals, NIBM, Penang 11700, Malaysia

**Keywords:** poly(3-hydroxybutyrate-*co*-4-hydroxybutyrate), collagen peptide, aminolysis, surface functionalization

## Abstract

The quest for a suitable biomaterial for medical application and tissue regeneration has resulted in the extensive research of surface functionalization of material. Poly(3-hydroxybutyrate-*co*-4-hydroxybutyrate) [P(3HB-*co*-4HB)] is a bacterial polymer well-known for its high levels of biocompatibility, non-genotoxicity, and minimal tissue response. We have designed a porous antimicrobial silver SSD blend/poly(3HB-*co*-4HB)-collagen peptide scaffold using a combination of simple techniques to develop a scaffold with an inter-connected microporous pore in this study. The collagen peptide was immobilised via -NH_2_ group via aminolysis. In order to improve the antimicrobial performance of the scaffold, silver sulfadiazine (SSD) was impregnated in the scaffolds. To confirm the immobilised collagen peptide and SSD, the scaffold was characterized using FTIR. Herein, based on the cell proliferation assay of the L929 fibroblast cells, enhanced bioactivity of the scaffold with improved wettability facilitated increased cell proliferation. The antimicrobial activity of the SSD blend/P(3HB-*co*-4HB)-collagen peptide in reference to the pathogenic Gram-negative, Gram-positive bacteria and yeast *Candida albicans* exhibited SSD blend/poly(3HB-*co*-4HB)-12.5 wt% collagen peptide as significant construct of biocompatible antibacterial biomaterials. Thus, SSD blend/P(3HB-*co*-4HB)-collagen peptide scaffold from this finding has high potential to be further developed as biomaterial.

## 1. Introduction

**Figure 1:**
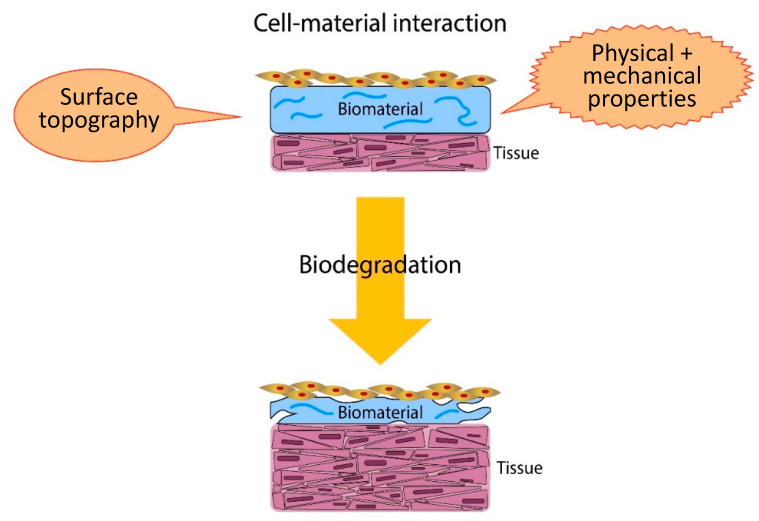
Schematic represents the crucial factors in tailoring a suitable biomaterial for tissue engineering.

Biomaterials serve as an interface with the biological systems to evaluate, augment, treat, or replace any tissue, organ or function of the body [1]. The success of biomaterials depends on several factors that determine their appropriate interaction with the host biological environment. Their physical and chemical properties, as well as topography and chemistry of the surface, have an important influence on cellular response [2]. In general, surfaces act as crucial platform in biology and medical fields since most of the biological reactions occur at the surface and interfaces [3]. In fact, the development of biomaterials requires detailed understanding on the interaction among the biomaterial surface, host tissue cells and extracellular matrix (ECM) to elicit ideal tissue response (Figure 1) [4].

An ideal biomaterial surface is necessary to stimulate a constructive cell response for wound reparation and tissue engineering, while unfavourable surface may indicate the material is to be explanted [5]. Biomaterial implanted in living organisms can trigger acute and chronic inflammatory responses [6]. Therefore, the right choice of material is crucial in tailoring biomaterial for tissue engineering. Among the various types of material, P(3HB-*co*-4HB) has gained attention for medical uses and regeneration medicine applications owing to its biocompatibility, chemical-diversity, biodegradability, non-genotoxicity and minimal tissue response in vivo [7]. Other biopolymers which have been widely used for biomaterials includes polyglycolic acid (PGA), poly(L-lactic acid) (PLLA) and poly(DL-lactic-*co*-glycolic acid) (PLGA), poly(l-lactic acid-*co*-ε-caprolactone) (PLCL) and polyurethane. However, the degradation of these biopolymers has proved to be a major drawback in medical applications. These biopolymers degrade acidic material and accumulate in the body. Thus, induces severe inflammatory responses, even causing cell and tissues necrosis [8]. 

However, the surface of P(3HB-*co*-4HB) is hydrophobic with minimal recognition sites for cell attachment. As such, surface modifications are an effective approach in designing scaffolds in order to achieve biocompatibility properties in tissue engineering applications [8]. Surface modifications involve changes only at the outermost surface orientation and composition of biomaterial, without affecting its bulk properties [9]. Extensive research has been conducted to prepare various surfaces using several methods of surface modifications including biological modifications where natural polymer such as protein, polysaccharides, collagen and cellulose has been extensively utilized in tissue engineering applications. Among these, collagen has been widely used in various biomedical applications due to its abundance in the ECM, non-immunogenicity and various sources of existing isolation methods. Likewise, the collagen fibres also have some unique structural properties which play an important role in stimulating adjacent cells that regulate functional response and tissue engineering [10].

In addition, it has been shown that development of antimicrobial scaffolds is garnering interest for being able to prevent possible infections therefore, used as an alternative to control infection and minimize the use of antimicrobial agents. Thus, this work exhibits the modification of the surface of biodegradable P(3HB-*co*-4HB) combined with antimicrobial agent silver sulfadiazine (SSD) and collagen peptide in order to develop and design promising antimicrobial and biocompatible scaffolds for biomedical applications. The surface modification of the SSD blend/P(3HB-*co*-4HB) was done using a combination of surface functionalization methods which includes a combination of salt leaching and the freeze-drying technique. The porous surface of P(3HB-*co*-4HB) was grafted with collagen peptide via aminolysis. Herein, the scaffolds were characterized by water contact angle, FTIR, SEM and subjected to cell proliferation assay using fibroblast cell L929. Increasing number of infections caused by pathogenic bacteria has severely affected human society these days. As such, the development of antimicrobial SSD blend/P(3HB-*co*-4HB)-collagen peptide is considered a significant reference for the construction of advanced biocompatible antibacterial biomaterials. 

## 2. Materials and Methods 

### 2.1. Fabrication of Porous SSD Blend/P(3HB-co-4HB) Aminolysed Collagen Peptide Scaffolds

**Figure 2:**
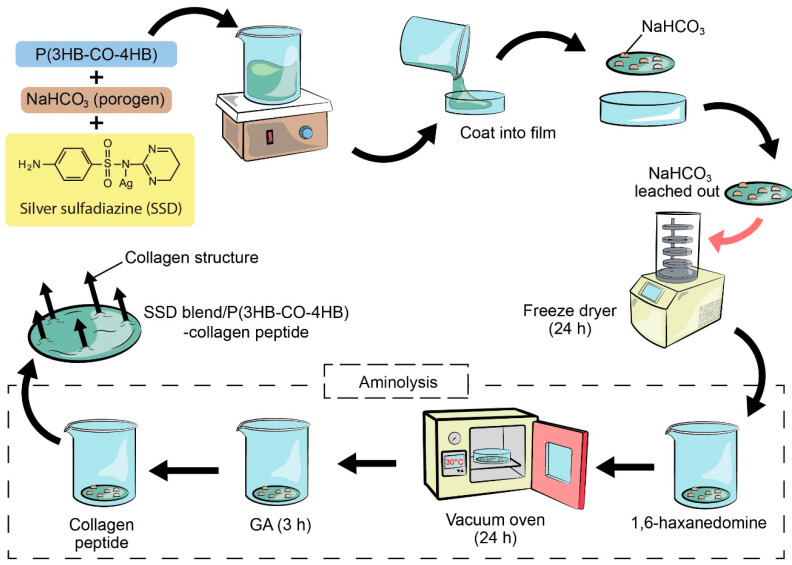
The summarised process of fabricating SSD blend/P(3HB-*co*-4HB)-collagen peptide.

The P(3HB-*co*-95 mol% 4HB) copolymer was synthesized as previously described [11]. The extracted copolymer was used to fabricate porous P(3HB-*co*-4HB) scaffolds by employing salt leaching and freeze-drying method. Briefly, an amount of 0.83 g of P(3HB-*co*-4HB) was dissolved in chloroform with SSD (4000 µg/mL). The mixture was stirred vigorously. Then, sodium bicarbonate (NaHCO_3_) which serves as porogen was added and stirred homogenously for 1 h. The solution was cast in glass Petri dish (diameter of 9 cm). The scaffolds were washed and the porogens were leached out. The scaffolds were further freeze-dried (24 h) and later vacuum-dried for 48 h using vacuum oven (BINDER GmbH, Tuttlingen, Germany) to remove any remaining solvent. The scaffolds were rinsed with a large amount (~200 mL) of deionized water to remove any impurities. Then it is immersed in 10 wt% 1,6-hexanediamine/2-proponal solution at 37 °C for 20 min [12], rinsed with deionized water for 24 h at room temperature to remove free 1,6-hexanediamine and finally dried in vacuum at 30 °C for 24 h to constant weight. This NH_2_-P(3HB-*co*-4HB) scaffold was then immersed in 1 wt% GA solution for 3 h at room temperature, followed by rinsing with a large amount (~200 mL) of deionized water for another 24 h to remove free GA. The scaffold was incubated in various concentrations of collagen peptide solution (pH 3.4) for 24 h at 2–4 °C. The collagen-P(3HB-*co*-4HB) membranes were rinsed with 1% acetic acid solution for 24 h to remove free collagen, followed by three washes with deionized water. The method of fabrication was summarized in Figure 2. The efficiency of collagen immobilization was determined as follows:

(1) $$\mathrm{Collagen}\;\mathrm{retain}=\left(W_{c}-W_{b}\right)-\left(W_{c}-W_{o}\right)/W_{c}\times 100\%$$

W_o_: Net weight scaffold without immobilisation of collagen peptide; W_b_: Net weight scaffold after immobilisation of collagen peptide; W_c_: Net weight scaffold without immobilisation of collagen peptide.

### 2.2. Characterization of Porous SSD Blend /P(3HB-co-4HB)-Collagen Peptide

The functional group of porous SSD Blend /P(3HB-*co*-4HB)-collagen peptide was analysed using Fourier transform infrared (FTIR) spectroscopy analysis (Model RX1, PerkinElmer, UK). The spectra were recorded in transmittance mode and with the wavenumber range of 650–4000 cm^−1^ [13].

The surface morphology was observed by scanning electron microscopy SEM (Leo Supra 50 VP Field Mission SEM, Carl-Ziess SMT, Oberkochen, Germany). The samples were prepared by coating with gold to improve conductivity of the beam.

The water uptake ability of scaffolds was calculated based on the differences in the weight of the scaffolds. Basically, the scaffolds were cut into 1 cm × 1 cm and dry weight before immersion (m_o_) and wet weight after immersion (m_f_) for 24 h and water uptake was calculated using the formula below:

Water uptake = (m_o_ − m_f_)/m_o_ × 100% (2)

In order to determine the wettability properties, water contact angle of the scaffolds was conducted using sessile drop method (KSV CM200 Contact Angle, KSV CAM 200, KSV Instruments Ltd., Helsinki, Finland). The scaffolds were cut into 1 cm × 1 cm pieces. The scaffolds were placed on the instrument and droplet of water was then deposited on the polymer surface by a specialized micro-syringe. The water droplet was observed from the computer screen and the contact angle was calculated.

The pore size and porosity measurement of the scaffolds was calculated using Image Analyzer Software (Olympus Co. Ltd., Tokyo, Japan). The values of 100 different spots were analyzed and averaged [14].

### 2.3. Antimicrobial Assay

The antimicrobial activity assay was carried out using four opportunistic bacterial strains which includes Gram-positive [(*Staphylococcus aureus* (ATCC 12600)], Gram-negative [*Pseudomonas aeruginosa* (ATCC 17588)] and yeast *Candida albicans* was obtained from Microbiology Lab, School of Biological Sciences, Universiti Sains Malaysia. The bacterial suspension (20 µL) with a total of 7.5 × 10^5^ CFU/mL was added onto porous SSD blend /P(3HB-*co*-4HB)-collagen peptide. All the scaffolds the scaffolds were sterilized under UV (Spectrolinker™, XL-1000UV Cross linker, New York, NY, USA) at 1200 µJ/cm^2^ for 30 min prior to testing. The scaffolds were incubated in the suspension at intervals of 0, 6, 12, and 24 h. At every interval, the suspension was spread on nutrient agar to observe the colonization of bacteria [15]. The percentages of inhibition were calculated using the following equation:

(3) $$C\%=\left(C_{o}-C_{e}\right)/C_{o}\times 100\%$$

where C% is percentage of inhibition, C_e_ is CFU after incubation period, and C_o_ is initial CFU before incubation period.

### 2.4. Cell Proliferation Assay

The proliferation of mouse fibroblast cell (L929, ATCC) on SSD blend /P(3HB-*co*-4HB)-collagen peptide was studied. The L929 cells were seeded on the scaffolds with a concentration of 2.5 × 10^4^ cells/mL in Modified Eagle Medium (MEM) supplemented with 2 mM L-glutamine, 1.5 g/L sodium bicarbonate, 1 mM of sodium pyruvate, 1000 U/mL penicillin-streptomycin and 10% (*v*/*v*) of bovine calf serum incubated at 37℃ in 5% (*v*/*v*) CO_2_ for 2-3 days. Prior to that the scaffolds were sterilized under UV (Spectrolinker™, XL-1000UV Cross linke, New York, NY, USA) at 1200 µJ/cm^2^ for 30 min [12]. The cells viability and proliferation were assayed with MTS [3-(4,5-dimethylthiazol-2-yl)-5-(3-carboxymethoxyphenyl)-2-(4-sulfophenyl)-2H-tetrazolium/PMS (phenazinmethosulfate) [16,17].

### 2.5. Statistical Analysis

All the data were analysed using ANOVA and Tukey’s HSD test (SPSS 20 software, SPSS 20 software, IBM, New York, NY, USA) and expressed as mean ± standard deviation (s.d.). The significance level of *p*-value < 0.05 was considered significant.

## 3. Results and Discussions

### 3.1. SSD Blend/P(3HB-co-4HB) Aminolysed Collagen Peptide

The porous scaffold with impregnated SSD as antimicrobial properties was prepared using a combination of salt leaching and freeze-drying technique. As a matter of fact, the weight ratio of SSD to polymer is 1:500. The final content of the SSD is 6.3 µg in the 1 cm^2^ scaffold. Subsequently, collagen peptide was immobilized onto the porous surface via aminolysis. The thickness of the scaffold was recorded at 626.0 µm ± 14. Theoretically, salt-leaching is a simple technique where desirable pore sizes were created by flushing the porogen with water, leaving behind pores formed by the porogen [18]. With this aim in mind, NaHCO_3_ was used as porogen due to ease of removal since it is soluble in aqueous media. Furthermore, trapped NaHCO_3_ is not harmful to the human body owing to its function as a buffering agent in the human body [19]. Freeze-drying is another commonly used method to form porous or micro-rough surface on scaffolds. Apart from that, freeze-drying technique produces a uniform porous structure for potential scaffolds in tissue engineering applications. The inter-pore openings and pore shape of the scaffolds were fabricated through the salt-leaching technique that is known to be uncontrollable. Hence, combining salt-leaching with other techniques may enhance and improve the pore inter-connectivity and networking [8,20].

**Figure 3:**
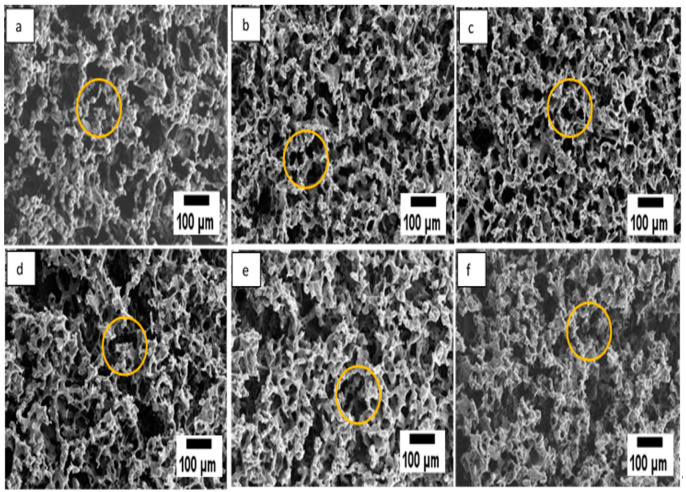
SEM micrographs of (**a**) P(3HB-*co*-4HB), (**b**) P(3HB-*co*-4HB)/2.5 wt% collagen peptide, (**c**) P(3HB-*co*-4HB)/5 wt% collagen peptide, (**d**) P(3HB-*co*-4HB)/7.5 wt% collagen peptide, (**e**) P(3HB-*co*-4HB)/10 wt% collagen peptide and (**f**) P(3HB-*co*-4HB)/12.5 wt% collagen peptide. The circles indicate the interconnected micro-pores.

The porosity of the developed SSD blend/P(3HB-*co*-4HB)-collagen peptide scaffolds were analysed through SEM technique as shown in Figure 3. The observation of SEM images exposed SSD blend/P(3HB-*co*-4HB)-collagen peptide surface comprised open pores surface. The results show that the porosity of the varied collagen concentration of aminolysed scaffolds are almost similar which are in the range 38 µm to 40 µm. The morphology of the surface revealed that, by combining salt-leaching and freeze-drying, a homogenous and interconnected macro-porous structure was produced.

Basically, the porous-based connectivity surface had enhanced the ECM architecture and provided a larger space to induce cell-material interactions [21,22].

**Figure 4:**
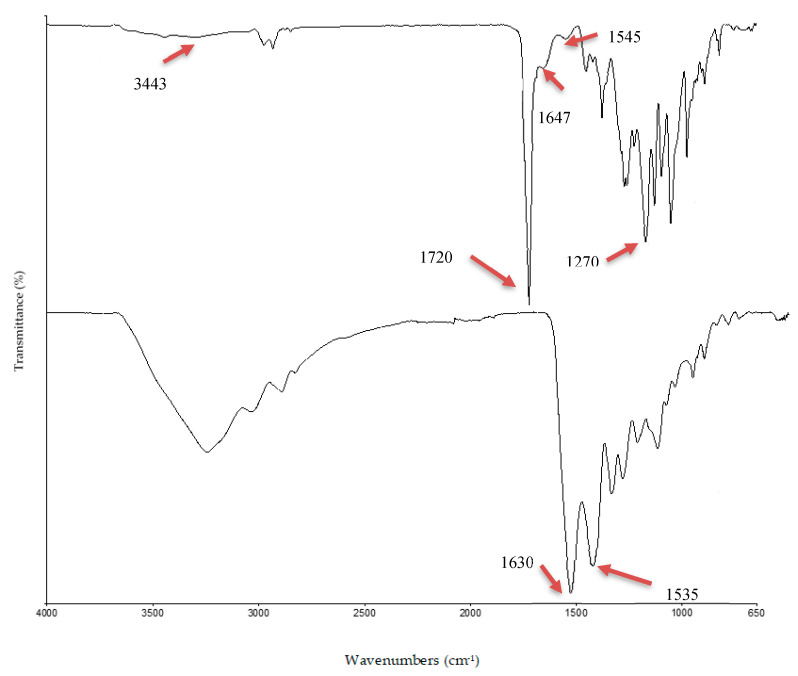
FTIR spectra of SSD blend/aminolysed P(3HB-*co*-4HB) scaffolds (**a**) collagen, (**b**) P(3HB-*co*-4HB)/collagen peptide.

As it is, aminolysis was carried out using 1,6-hexadiamine in 2-propanol; whereby the amino group of 1,6–hexadiamine reacts with carbonyl group of P(3HB-*co*-4HB). The P(3HB-*co*-4HB)-NH_2_ scaffold was treated with gluteraldehyde (GA) in order to immobilize the collagen peptide onto the P(3HB-*co*-4HB) surface. The scaffolds were then immobilised using varied collagen concentrations (2.5 wt%, 5 wt%, 7.5 wt%, 10 wt%, 12.5 wt%), respectively and this was verified using FTIR (Figure 4).

The FTIR was used to further confirm the immobilization of collagen peptide on to SSD blend/P(3HB-*co*-4HB) scaffolds. Based on the FTIR spectrums in Figure 4a, the collagen was identified based on the presence of amide I at 1630 cm^−1^, amide II band at 1531 cm^−1^. The conversion of primary amine to secondary amine by the formation of C=N with the elimination of -NH_2_ was observed at 1647 cm^−1^ of aminolysed P(3HB-*co*-4HB)/collagen peptide scaffolds (Figure 4b). It also exhibited the absorption band at 1720 cm^−1^ present in the aminolysed P(3HB-*co*-4HB)/collagen peptide scaffolds corresponds to the ester carbonyl group (C=O), being the main functional group of P(3HB-*co*-4HB). This finding has confirmed that the collagen was successfully immobilized to the P(3HB-*co*-4HB) scaffolds [13]. The FTIR spectrum was also used to characterize the molecular structure of SSD blended into the scaffolds. The absorption peak of at 3443 cm^−1^ corresponds to -NH_2_ symmetric of SSD. In contrast, the peak at 1545 cm^−1^ belongs to the phenyl skeletal vibration od SSD. The peak at 1228 cm^−1^ characterized the stretching vibration of (SO_2_) group. This further emphasizes the presence of SSD blended into the aminolysed P(3HB-*co*-4HB)/collagen peptide scaffolds [23,24].

The actual amount of collagen peptides onto P(3HB-*co*-4HB) scaffolds were measured based on the weight of scaffolds. The amount of collagen immobilised onto SSD blend/collagen-peptide coated P(3HB-*co*-4HB) and SSD blend/collagen-peptide coated P(3HB-*co*-4HB) surface was manifested in Table 1. It can be deduced that the amount of collagen retained on the scaffolds was in the range of 78–85% without any significant difference among the scaffolds with different collagen concentration [13].

### 3.2. Hydrophilicity of SSD Blend/P(3HB-co-4HB)-Collagen Peptide Scaffold Wettability

**Figure 5:**
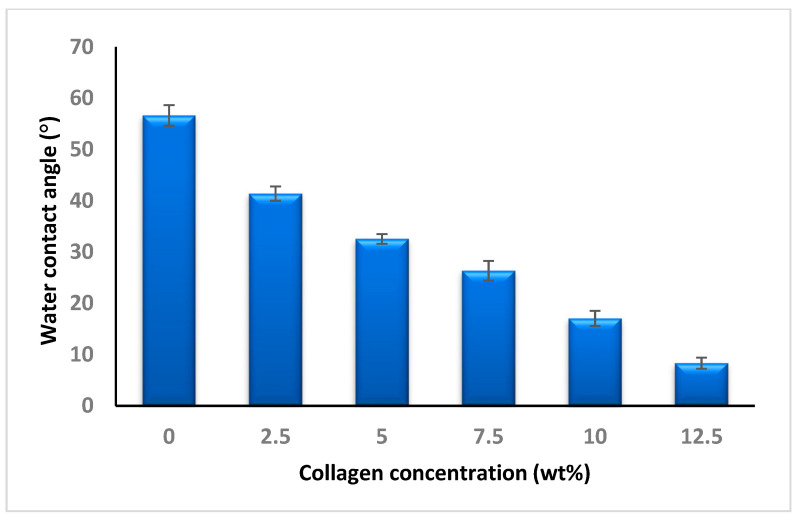
Water contact angle analysis of SSD blend/aminolysed P(3HB-*co*-4HB)-collagen peptide scaffold. Data represent means ± standard deviations (n = 3).

The hydrophilicity of SSD blend/P(3HB-*co*-4HB) aminolysed collagen peptide scaffold was determined using water contact angle analysis. Figure 5 presented the effects of water contact angle based on the interaction with various collagen peptide concentrations (0, 2.5, 5, 7.5, 10, 12.5 wt%). It can be deduced as the concentration of collagen peptide increased; the water contact angle of the scaffold decreased [13]. A four-fold of increase in hydrophilicity was observed from SSD blend/P(3HB-*co*-4HB) to the SSD blend/P(3HB-*co*-4HB) aminolysed 12.5 wt% collagen peptide. It is crucial to have an increased hydrophilic nature of biomaterial scaffolds surface as this can lead to improved cellular behaviors which includes cell adhesion, proliferation and differentiation. The improvement of SSD blend/P(3HB-*co*-4HB) aminolysed 12.5 wt% collagen peptide is comparable to nanofibers scaffolds of other biopolymers which includes PLGA, PLLA, PLCL and polyurethane as summarized in Table 2.

**Figure 6:**
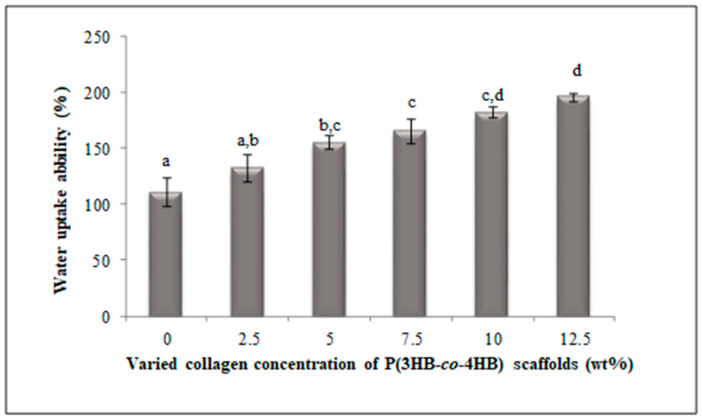
Water uptake ability of SSD blend/P(3HB-*co*-4HB) aminolysed collagen peptide scaffold. Data represent means ± SD (n = 3). Mean data accompanied by different alphabets (a-d) indicates significant difference with each respective group (Tukey’s HSD test, *p* < 0.05).

The water uptake ability analysis of the fabricated scaffolds was shown in Figure 6. Based on the results, the water uptake capacity increased as the concentration of immobilised collagen peptide in scaffold was increased [25]. The P(3HB-*co*-4HB) copolymer scaffolds recorded 111% of water absorption capacity, whereas the P(3HB-*co*-4HB)-collagen peptide with collagen peptide concentration ranging 2.5 to 12.5 wt% recorded water uptake ability between 132% to 196% following an increasing trend. As a matter of fact, the two-fold increase in water absorption capacity due to the immobilization of collagen peptide developed a hydrophilic P(3HB-*co*-4HB) copolymer scaffold.

P(3HB-*co*-4HB) presents a hydrophobic nature, with poor hydrophilicity which is one of the limitations for biomedical application. Therefore, introducing collagen peptide via -NH_2_ groups using aminolysis was expected to introduce more hydrophilic contact points. Based on the results obtained, it can be observed that an increase in collagen peptide concentration up to 12.5 wt% resulted in an increase in surface wettability. Thus, indicating the increase of hydrophilicity at higher concentrations. Therefore, aminolysis was effective in modifying the hydrophilicity of P(3HB-*co*-4HB) surface [24,25].

### 3.3. Biocompatibility and Cell Proliferation of SSD Blend/P(3HB-co-4HB) Aminolysed Collagen Peptide Scaffolds

**Figure 7:**
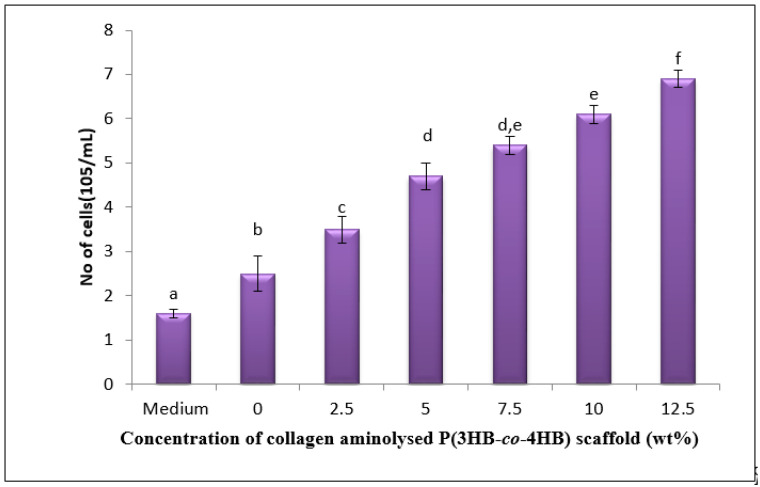
Proliferation of L929 cells on the various fabricated SSD blend/P(3HB-*co*-4HB) aminolysed collagen peptide scaffolds. Data represent means ± SD (n = 5). Mean data accompanied by different alphabets (a-f) indicates significant difference with each respective group (Tukey’s HSD test, *p* < 0.05).

The cellular behaviour or known as cell growth were assessed by seeding L929 fibroblasts cells onto the various aminolysed fabricated P(3HB-*co*-4HB) collagen peptide scaffolds. As illustrated in Figure 7, the number of fibroblast cells found to have a stable growth on all the tested scaffolds over the time period of 3 days compared to the initial seeding. The fusion of collagen was able to improve the cytocompatibility. Surface modification via aminolysis has been commonly employed to enhance cell proliferation without property alteration [12,30].

Concerning the increasing drift along the concentrations, the highest cell proliferation rate was acquired on scaffolds with 12.5 wt% collagen with cell count of 6.9 × 10^5^ cells/mL. This is in accordance with highest water contact angle (8.3°) leading to better hydrophilicity of P(3HB-*co*-4HB) at 12.5 wt% collagen blend scaffolds. Improved wettability facilitates cell seeding on aminolysed scaffolds as a five-fold increase cell proliferation was observed in the the SSD blend/P(3HB-*co*-4HB) aminolysed 12.5 wt% collagen peptide [31].

**Figure 8:**
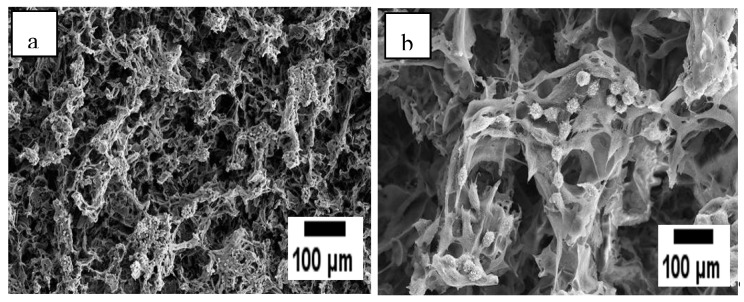
Proliferation of L929 cells on the SSD blend/P(3HB-*co*-4HB) scaffolds aminolysed 12.5 wt% collagen peptide. Magnification of 100× (**a**) and 503× (**b**) were used.

On top of it, SEM analysis of P(3HB-*co*-4HB) scaffold (Figure 8), further supported the efficiency of 12.5 wt% collagen blend in terms of the surface morphology which happened to be rough and porous exhibiting the highest proliferation rate. The extracellular matrix (ECM) proteins, such as collagen peptides, adsorbed on to the scaffold surface when exposed to the physiological environment, allowing indirect fibroblast-scaffold interaction via the adsorbed ECM proteins. The cell-protein interaction of the scaffold is regulated by integrin, a cell membrane receptor [32]. The cellular activity criteria of SSD-blend/aminolysed P(3HB-*co*-4HB) scaffolds indicated their suitability for biomaterial applications.

### 3.4. Antimicrobial Analysis of Fabricated SSD Blend/P(3HB-co-4HB) Aminolysed Collagen Peptide Scaffold.

**Figure 9:**
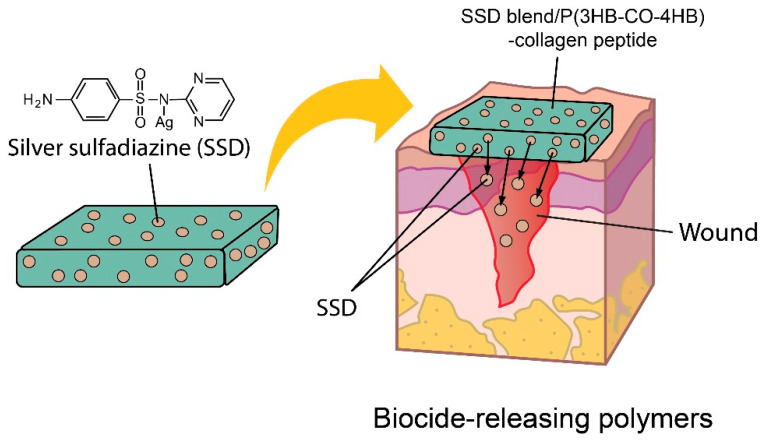
Schematic represents the SSD impregnation aminolysed scaffolds as biocide-releasing polymers with sustained release of SSD which efficiently kill microbes in contact.

The antimicrobial activity of the SSD blend/P(3HB-*co*-4HB)-collagen peptide against the microorganisms was quantitatively assayed through the colonization method. The data (Table 3) obtained from the colonization method revealed that the SSD exhibited good antimicrobial properties with variability in the inhibition rate against different tested strains. Meanwhile, the antimicrobial P(3HB-*co*-4HB) aminolysed collagen peptide scaffold showed susceptibility for *Staphylococus auerus*, *Pseudomonas aeruginosa* and *Candida albicans* at 24 h where their inhibition rate reached 100% at 48 h. The results obtained exhibited that SSD showed varied inhibitory rates against the tested microorganisms. SSD impregnation aminolysed scaffolds are biocide-releasing polymers with prolonged release of SSD to efficiently kill microbes on contact. The sustained release of silver ions adheres to the microbial cell surface and causes membrane damage which enables the silver ions to penetrate inside the microbial cells and interact with cellular organelles and biomolecules, thereby, affecting respective cellular machinery causing cell death (Figure 9). Bacterial cells and yeast have a negative net charge at the surface due to their membrane proteins, as such the Ag+ ion is attracted and able to disrupt the cytoplasmic membrane, thus causing cell lysis [33]. Herein, the incorporation of antimicrobial compounds onto polymer fibrous scaffolds with further antimicrobial properties release can be further improved as drug delivery system and regenerative medicine. The SSD blend/P(3HB-*co*-4HB)-collagen peptide has the potential of prolonged release of SSD, which provided an improved antimicrobial effect [34,35,36].

## 4. Conclusions

The SSD-blend/P(3HB-*co*-4HB) aminolysed collagen peptide scaffold with porous surface was successfully developed. Collagen conjugation was done through the aminolysis technique where collagen peptide was covalently linked to P(3HB-*co*-4HB) scaffold. These P(3HB-*co*-4HB) scaffolds exhibited enhanced bioactivity of fibroblast cell (L929) growth and increased hydrophilicity with the presence of collagen peptides in the scaffold matrices. Scaffolds with physical modification effectively allow physical entrapping of therapeutic protein within a polymeric device. Thus, the immobilization of collagen provides a simple and convenient way to incorporate biological molecules onto polymer scaffolds, which makes this scaffold suitable for tissue engineering such as bone, cartilage, ligament, skin, vascular tissues, neural tissues, and skeletal muscle. Moreover, the main challenge in biomedical applications is developing scaffolds that promote tissue–cell interactions, such as adhesion and proliferation, while simultaneously inhibiting bacterial colonization. Therefore, the SSD-blend/P(3HB-*co*-4HB) aminolysed collagen peptide scaffold would be useful for bone, cartilage, and tissue regeneration with antimicrobial activity that can be released in a prolonged manner due to the presence of a reversible thermodynamic equilibrium between immobilized collagen and the incorporated antimicrobial agent.

## Figures and Tables

**Table 1:** The collagen peptide immobilisation efficiency based on the percentage of collagen reatined on the SSD blend/aminolysed P(3HB-*co*-4HB) scaffolds.

Polymer	Amount of Collagen Retained (%)
P(3HB-*co*-4HB) + 2.5 wt% collagen	78 ± 2 ^a^
P(3HB-*co*-4HB) + 5 wt% collagen	80 ± 3 ^a,b^
P(3HB-*co*-4HB) + 7.5 wt% collagen	82 ± 3 ^a,b^
P(3HB-*co*-4HB) + 10 wt% collagen	83 ± 2 ^a,b^
P(3HB-*co*-4HB) + 12.5 wt% collagen	85 ± 1 ^b^

Data represent means ± SD (n = 3). Mean data accompanied by different alphabets (a-b) indicates significant difference with each respective group (Tukey’s HSD test, *p* < 0.05).

**Table 2:** Comparison of the water contact angle of biopolymer scaffolds widely used as biomaterials from previous studies with the developed SSD blend/ aminolysed P(3HB-*co*-4HB)-collagen peptide scaffolds against various microorganisms.

Biopolymer/Materials	Water Contact Angle Results	References
PLGA/collagen nanofibers	It is recorded that the contact angle of this electrospun PLGA/collagen scaffold is 70° and the addition of collagen increased the wettability of the PLGA to be developed as tissue engineering scaffolds for regenerative medicine	[26]
PLLA/collagen nanofibers	The incorporation of collagen to the PLLA biopolymer nanofibers to improve the surface wettability resulted in a water contact angle of 80° for the development of bone graft.	[27]
Polyurethane/collagen by green facile method	The addition of collagen to the polyurethane exhibited water contact angle between 80–85° for the development of smart materials that are environmentally friendly and easier to process	[28]
collagen/PLCL nanofibers	The PLCL electrospun nanofiber with collagen had an increased wettability between 0° (10 s) and 24° (5 s) for the development of desirable scaffold for	[29]
P(3HB-*co*-4HB) aminolysed collagen peptide	The porous P(3HB-*co*-4HB) scaffolds incorporated with collagen in this study showed an increased surface wettability with water contact between 4.3° ± 1.8° − 43.7° ± 1.7° which can be further developed as tissue regenerative scaffolds	Current paper

**Table 3:** Antimicrobial test of SSD blend/ aminolysed P(3HB-*co*-4HB)-collagen peptide scaffolds against various microorganisms.

Time (h)	Inhibition of Microorganisms (%)		
	*Staphylococus auerus* ATCC 12600	*Pseudomonas aeruginosa* ATCC 17588	*Candida albicans*
0	0 ± 0	0 ± 0	0 ± 0
6	24 ± 5	25 ± 7	32 ± 2
12	45 ± 3	55 ± 7	61 ± 8
24	65 ± 9	92 ± 5	82 ± 8
48	100 ± 0	100 ± 0	100 ± 0

Values are mean ± SD of three replicates.
